# Construction and Activity Testing of a Modular Fusion Peptide against *Enterococcus faecalis*

**DOI:** 10.3390/antibiotics12020388

**Published:** 2023-02-14

**Authors:** Salim Manoharadas, Mohammad Altaf, Naushad Ahmad, Abdulwahed Fahad Alrefaei, Basel F. Al-Rayes

**Affiliations:** 1 Department of Botany and Microbiology, College of Science, King Saud University, P.O. Box 2454, Riyadh 11451, Saudi Arabia; 2 Central Laboratory RM 63AA, College of Science, King Saud University, P.O. Box 2454, Riyadh 11451, Saudi Arabia; 3 Department of Chemistry, College of Science, King Saud University, P.O. Box 2454, Riyadh 11451, Saudi Arabia; 4 Department of Zoology, College of Science, King Saud University, P.O. Box 2454, Riyadh 11451, Saudi Arabia

**Keywords:** enzybiotics, bacteriophage, *Enterococcus faecalis*, antibiotic-resistant bacteria

## Abstract

The emergence of antibiotic resistance in enterococci is a great concern encountered worldwide. Almost all enterococci exhibit significant levels of resistance to penicillin, ampicillin, semi-synthetic penicillin and most cephalosporins, primarily due to the expression of low-affinity penicillin-binding proteins. The development of new and novel antibacterial agents against enterococci is a significant need of the hour. In this research, we have constructed a modular peptide against *Enterococcus faecalis*. The enzymatic domain of the constructed peptide BP404 is from the bacteriocin BacL1 and the cell wall binding domain from endolysin PlyV12 of phage ϕ1. The protein BP404 was found to be active against two tested strains of *Enterococcus faecalis*, with a reduction in cell density amounting to 85% and 65%. The cell wall binding assay confirms the binding of the protein to *Enterococcus faecalis*, which was not seen towards the control strain *Escherichia coli*, invariably pointing to the specificity of BP404. To the best of our knowledge, this is one of the first instances of the development of a chimeric peptide against *Enterococcus faecalis*. This study points out that novel proteins can be genetically engineered against clinically relevant enterococci.

## 1. Introduction

*Enterococcus faecalis* has been emerging as one of the most significant opportunistic pathogens associated with hospital acquired infections [1]. *E. faecalis* causes various nosocomial infections such as meningitis, bacteremia, neonatal infections, wound infections, and urinary tract infections. In addition, *E. faecalis* is also reported to be associated with biofilm infections, particularly caused by implanted medical devices [2]. *E. faecalis* infections are also very common in dental diseases such as periimplantitis, caries and periodontitis [3,4]. Alarmingly, the emergence of antibiotic resistant strains of *E. faecalis* complicates the treatment of infections caused by *E. faecalis*. Antibiotic resistance in enterococci has been drastically increasing since 1980s, with vancomycin resistant enterococci being the latest addition to the list [5,6]. There are certain virulence factors that enable the *Enterococcus* sp., to colonize surfaces and promote biofilm formation [7,8]. The adherence of *Enterococcus* sp., to eukaryotic cells is also promoted by the aggregation substance (AS) [9]. One of the major virulence factors, the Esp protein, contributes to antimicrobial resistance by facilitating the interaction of *E. faecalis* to primary surfaces and subsequent formation of biofilms [10,11].

Certain peptides, or bacterial proteins that display antimicrobial activities, are known as bacteriocins [12]. There are two types of bacteriocins described in bacteria, class I and class II. Bacteriocins belonging to class I are usually created by post-translational modifications and contain nonproteinogenic amino acids [13]. Only enterocin W and β-hemolysin have been identified in *Enterococcus* sp. as class I bacteriocins [14,15]. Most of the bacteriocins from *E. faecalis* and *E. faecium* belong to class II and are non-modified antimicrobial peptides, including enterocin A, AS-48, Bac21, Bac31 and many others [16,17,18]. Apart from the heat and acid, stable low molecular weight bacteriocins classified as class I and class II bacteria also encompass heat liable antimicrobial proteins, which possess enzymatic bactericidal activity known as bacteriolysins [19]. The bacteriolysins in enterococcus includes bacteriocin 41 (Bac41) and enterolysin A [20,21]. Bac41 operon comprises of six functionally distinct open reading frames. The antibacterial activity of Bac41 is rendered by two ORF products, BacL_1_ and BacA. Even though secreted individually, both these proteins act in a coordinate manner to exert activity against *E. faecalis* peptidoglycan [21]. BacL_1_ is an endopeptidase that enzymatically cleaves the d-isoglutamyl-l-lysine bonds in *E. faecalis*, when the co-factor BacA is present [22]. BacL_1_ also possess a triple repeat of the sh3 domain located in the c-terminal region, comprising 261 amino acids. It is also interesting that BacL_1_ displayed its activity only against *E. faecalis* strains in the log phage of growth and not in stationary phase [18]. 

Bacteriophage based lytic enzymes capable of degrading bacterial cell walls and causing cell death is called enzybiotics, which is a hybrid term linking ‘enzyme’ and ‘antibiotic’ [23]. The term enzybiotics has gained more coverage in recent times, typically referring to almost all natural or recombinant peptides displaying anti-bacterial activity. This list includes bacteriocins, endolysins and even antifungal enzymes of natural origin [24]. Endolysins, synthesized at the end of the phage lytic cycle, target different bonds in the cell wall of bacteria. They can be broadly classified based on the enzymatic action displayed: (i) endopeptidases (ii) N-acetyl-β-d-glucosaminidase (iii) N-acetylmuramoyl-l-alanine amidases (iv) lytic transglycosylases and (v) lysozymes (N-acetyl-β-d-muramidases) [25]. Over the last decade, there were many enzybiotics developed against bacteria, particularly against Gram-positive bacteria. They were found to exert antibacterial activity either individually or in combination with antibiotics [23,26]. Endolysins are usually found as in modular structure. Modular structure is composed of individual domains with separate functions. One or more domains function to enzymatically cleave bonds in the cell wall, whereas the cell wall binding domain is usually found as a single domain which facilitates the specific binding of the endolysin to the substrate, such as teichoic acids or carbohydrate moiety in the cell wall [27,28].

PlyV12 is an endolysin encoded by the phage Φ1 which infects *E. faecalis*. Phage Φ1 belongs to the myoviridae family of phages which possess a contractile tail and icosahedral head [29]. Purified phage Φ1 endolysin, PlyV12 was earlier shown to display substantial exolytic activity against different strains of both *E. faecalis* and *E. faecium* [30]. In addition, the lysin also showed activity against several staphylococcal and streptococcal strains. It is speculated that the cell wall binding domain of PlyV12 targets a unique carbohydrate epitope in the cell wall that is common in these pathogenic bacteria [30]. 

In this work we aimed to create a novel enzybiotic BP404 by linking the catalytic domain of BacL_1_ with the cell wall binding domain of PlyV12. BP404 was found to be active against two tested strains of *E. faecalis*. To best of our knowledge, this is one among the first instance where a chimeric bacteriocin based enzybiotic has been engineered against *E. faecalis*.

## 2. Results

### 2.1. Construction of Protein BP404

**Figure 1:**
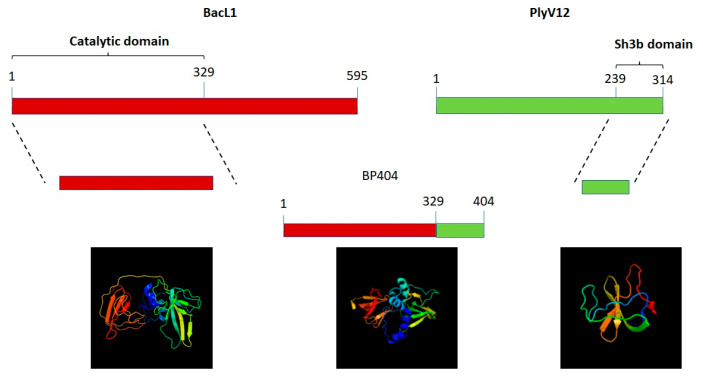
Construction of protein BP404 against *E. faecalis*. The catalytic domain from BacL_1_ was linked to the cell wall binding domain of PlyV12. The corresponding structure predicted by the phyre program is shown below.

Initially, the phyre program [31] was used to predict the structure of the catalytic domain of the BacL_1_ from *E. faecalis* and the cell wall binding domain of PlyV12 endolysin from the bacteriophage phage Φ1 and the recombinant chimeric protein, BP404 (Figure 1). As evident from the diagram, the catalytic domain of BacL_1_ comprised of the helix, coil and β strands. However, the cell wall binding sh3b domain of the endolysin PlyV12 did not possess any helix structure and was composed primarily of coil and β strands.

**Figure 2:**
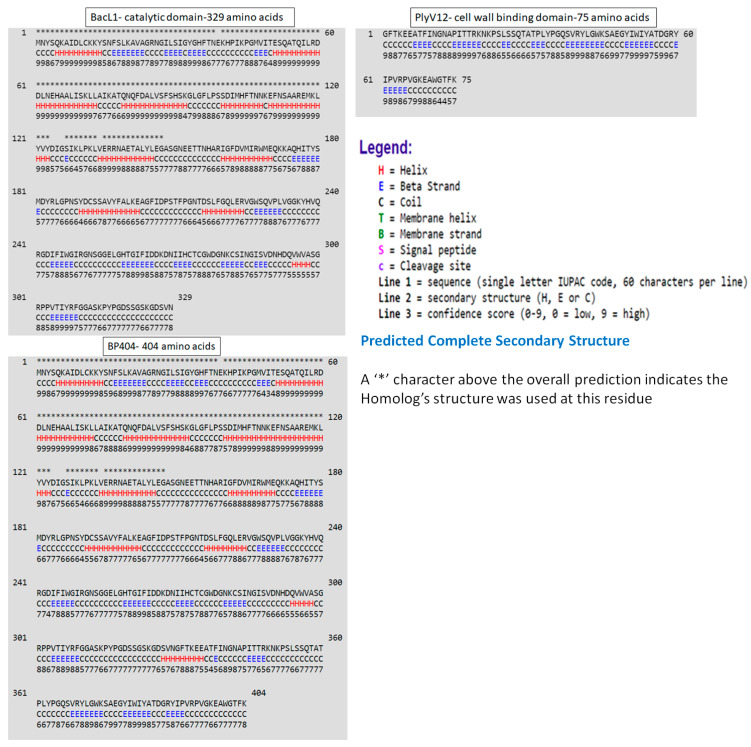
Secondary structure prediction of the protein domains. The secondary structure of the catalytic domain of BacL_1_ and the cell wall binding domain of PlyV12 is shown above. The secondary structure of the chimeric protein BP404 is shown below. ‘H’ represents helix, ‘E’ represents β strands and ‘C’ represent coil structure. The secondary structure was predicted by the proteus structure prediction server.

Furthermore, we wanted to confirm the structural configuration of the catalytic domain, sh3b domain and the recombinant protein BP404. The secondary structure prediction was done using the proteus structure prediction server [32] (Figure 2).

As seen in Figure 2, a predominant helix structure is found in the catalytic domain of BacL_1_, represented as ‘H’ in Figure 2, followed by the coil structure (C). β-strands (E) were found in a lower proportion in comparison with the other structures. In contrast, the sh3b domain from PlyV12 predominantly possess the coil and β-strands and no stretch of the helix pattern was found. BP404 protein, which was constructed by fusing the catalytic domain from BacL_1_ and sh3b domain from PlyV12 endolysin had the helix, coil and β-strands in the N-terminal region and the C-terminal region, harbored β-strands and coil structure. The fusion protein BP404 was overexpressed and was purified to homogeneity. The activity of the protein was tested against *E. faecalis* strains, as we speculate that the cell wall binding domain would bind to the cell wall of *E. faecalis* and the catalytic domain will cleave specific bonds in the *E. faecalis* peptidoglycan.

### 2.2. Synthesis and Purification of BP404

**Figure 3:**
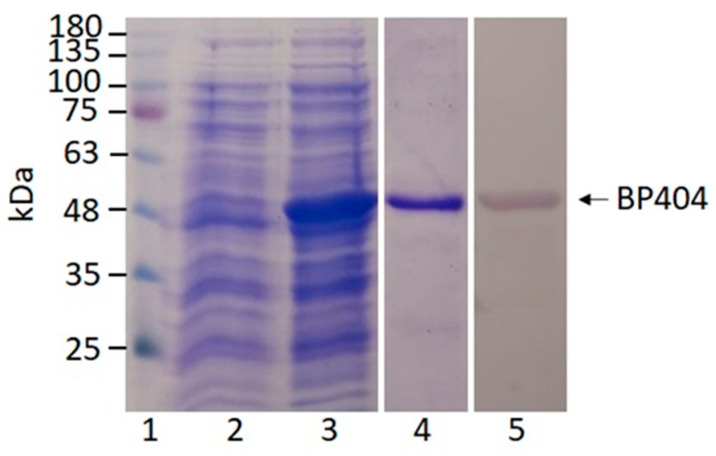
Expression, purification, and analysis of BP404 protein. Lane 1 shows the marker proteins. The un induced culture showed no synthesis of BP404 protein (lane 2). The IPTG induction of the gene BP404, synthesized the protein of the expected size (lane 3). The soluble protein was purified using Ni-NTA agarose. The purified protein resolved on the gel is shown in lane 4. The western blot performed to detect the protein is shown in lane 5.

The recombinant protein was synthesized by inducing the gene cloned under IPTG inducible promoter. The expression of the gene was performed at 16 °C. The protein synthesis was confirmed by resolving the uninduced and induced cultures on a 12% SDS PAGE gel. As shown in Figure 3, lane 2 represent the uninduced culture where no expression of the protein is seen. The size of BP404 protein along with the N-terminal linked 6x histidine tag is 46 kDa, which was seen after IPTG induction (Figure 3: Lane 3). The protein was purified using Ni-NTA by making use of the histidine tag fused to the N-terminal of protein BP404. The Ni-NTA bound protein was eluted and was dialyzed in buffer A. The purity of the dialyzed protein was evaluated by loading on to a 12% SDS PAGE gel. The purity of BP404 was estimated to be more than 90% (Figure 3: Lane 4). Western blot analysis was performed using the antibody against the 6x histidine tag found on the N-terminal of the protein, to rule out the possibility of any degradation products and to invariably test the purity of the protein. The western blot analysis revealed that the purified BP404 protein was pure with no degradation products (Figure 3: Lane 5). The purified BP404 protein was tested for antibacterial activity against two *E. faecalis* strains.

### 2.3. Protein BP404 Displays Activity against E. faecalis Strains

**Figure 4:**
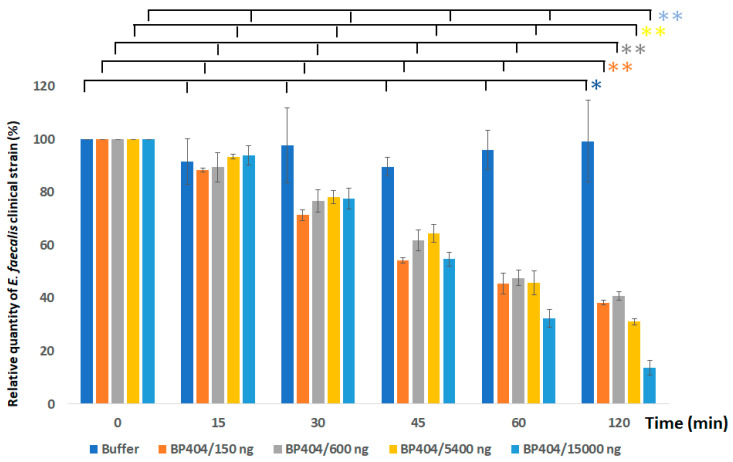
Activity testing of BP404 purified protein against *E. faecalis* clinical isolate. Varying concentrations of BP404 was tested against clinical strain of *E. faecalis*. The reduction in cell density is plotted as percentage (Y-axis) against the incubation time (X-axis). Error bars represent standard deviation. The statistical test of variance was calculated by one-way ANOVA (* *p* ≥ 0.05; ** *p* ≤ 0.05). The experiment was performed in triplicate.

Antibacterial activity of the constructed recombinant protein BP404 was tested against two strains of *E. faecalis*. Different concentrations of BP404 protein (150 ng, 600 ng, 5400 ng and 15,000 ng per ml) was added to 1 × 10^7^ CFU per ml of *E. faecalis* clinical isolate and ATCC 29212 strain in buffer A. After addition of the protein, the mix was incubated at 37 °C and the decrease in OD_600_ was analyzed over a period of 120 min. The protein exerted its antibacterial activity against both tested bacteria, with the activity ratio higher towards *E. faecalis* clinical isolate (Figure 4). As seen in Figure 4, the best activity was achieved when 15,000 ng of the purified BP404 protein was used. A decrease of 70% optical density was observed within 60 min following addition of 15,000 ng of BP404. The OD_600_ was further reduced by 85% after 120 min following the addition of the protein. Meanwhile, lower concentrations of the proteins (150 ng, 600 ng and 5400 ng) was also able to exert activity against *E. faecalis* clinical strain, albeit at a lower intensity. The reduction in OD_600_ of *E. faecalis* clinical strain was 60% with BP404 concentration of 150 ng and 600 ng after 120 min after treatment. When the buffer-treated control OD_600_ of *E. faecalis* clinical strain remained unchanged over the course of time, BP404 protein at a concentration of 5400 ng reduced the optical density by 70% by the end of 120 min following protein addition. The statistical significance was calculated with one-way ANOVA (https://goodcalculators.com/one-way-anova-calculator/ (5 February 2023). The data obtained for the buffer treated sample did not show a major degree of variance (* *p* ≥ 0.05). However, data from the BP404 treated bacterial samples was showing variance at all the different time points tested (** *p* ≤ 0.05).

**Figure 5:**
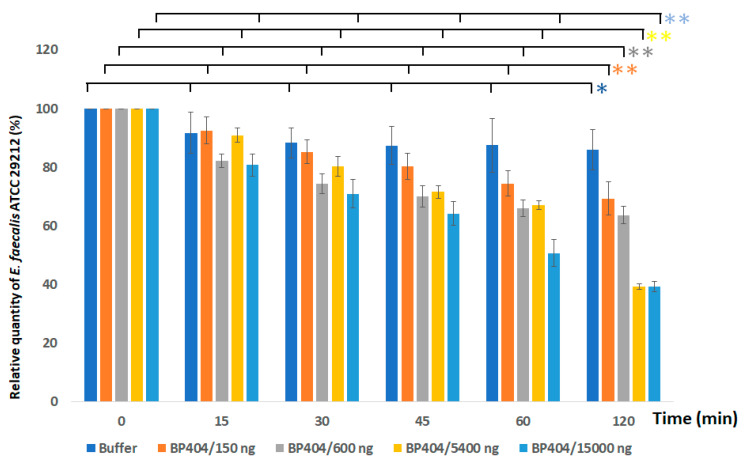
Activity testing of BP404 purified protein against *E. faecalis* ATCC 29212. Activity of the purified BP404 against *E. faecalis* ATCC 29212 was evaluated with different concentrations of protein. Error bars shows the standard deviation and the statistical variation was calculated by one-way ANOVA (* *p* ≥ 0.05; ** *p* ≤ 0.05). The experiment was performed in triplicate.

In comparison with the antibacterial activity exerted by BP404 against *E. faecalis* clinical strain, lower activity was observed against the *E. faecalis* ATCC 29212 strain. The highest activity was exerted by BP404 at a concentration of 15,000 ng, with the reduction in OD_600_ amounting to 50% within 60 min of incubation, which further proceeds to 60% reduction within 120 min (Figure 5). Interestingly, the reduction in OD_600_ with BP404 at a concentration of 5400 ng of protein had a similar activity as 15,000 ng at the end of 120 min following addition of the protein. A ten percent reduction in the OD_600_ was also observed with the buffer control. However, with the statistical test of variance, the buffer treated bacterial sample did not show data variance across the tested time points (* *p* ≥ 0.05). The statistical test of variance was performed using one-way ANOVA calculator. The BP404 treated bacterial samples showed variance at different time points, as assessed by *p*-value (** *p* ≤ 0.05). The data for *p*-value obtained from one-way ANOVA analysis is shown in Supplementary Figure S1.

To visualize the antibacterial activity of the BP404 protein against the tested *E. faecalis* strains, the highest concentration (15,000 ng) of the purified BP404 was added to the bacterial samples and confocal microscopy was done.

### 2.4. Live/Dead Staining of the E. faecalis Strains Treated with BP404

**Figure 6:**
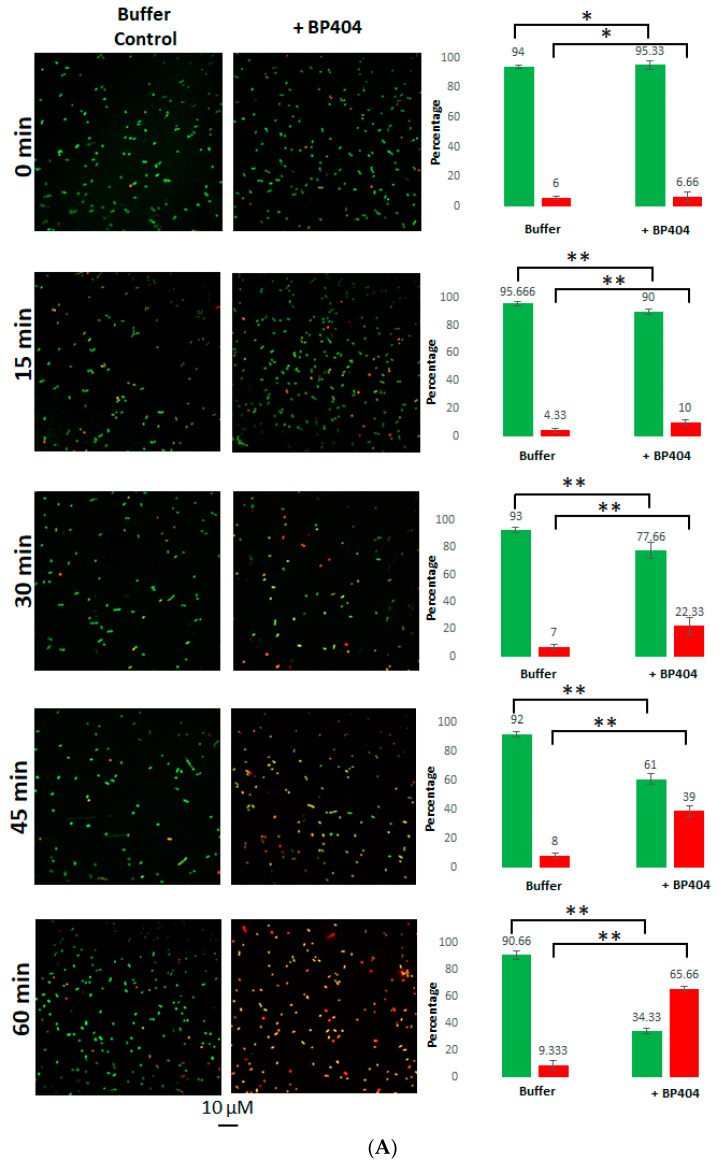

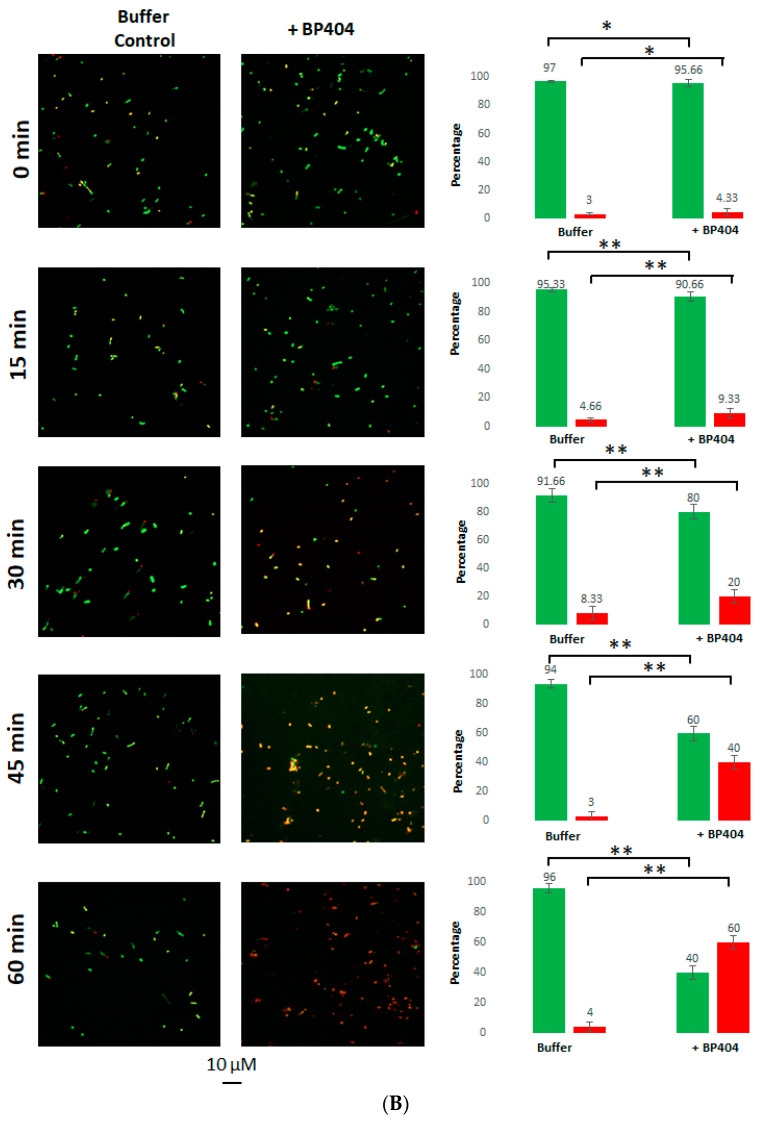
Live/Dead staining of *E. faecalis* strains treated with 15,000 ng of protein BP404 (**A**) Clinical isolate of *E. faecalis* was treated with BP404. Confocal images and the corresponding graph representing the percentage of live cells (green bars) and dead cells (red bars) is shown. (**B**) *E. faecalis* ATCC 29212 treated with the buffer or BP404 is shown. The live cells appear as green and dead cells appear as red in confocal imaging. The experiment was performed in triplicate and the standard deviation is shown by error bars. The statistical test of variance was calculated by one-way ANOVA (* *p* ≥ 0.05; ** *p* ≤ 0.05).

In order to confirm the antibacterial activity of protein BP404 against *E. faecalis* strains, a live/dead staining was performed after the addition of 15,000 ng of BP404 to 1 × 10^7^ CFU of bacteria. The ratio of live/dead bacteria was observed with confocal microscopy. As observed earlier, the BP404 was able to significantly reduce the *E. faecalis* clinical isolate. As seen in Figure 6A, in the buffer control treated samples, the live to dead ratio of cells was 9:1 after 60 min after treatment. In contrast, BP404 treated *E. faecalis* clinical isolate live to dead cell ratio was 0.5:1.9, which translates to only 34.33% live cells (in green) and 65.66% dead cells (in red). At 15 min after addition of the protein, only 10% of the cells were dead. This number increased to 23.33% and 39% at the end of 30 min and 45 min, respectively. 

The BP404 treated *E. faecalis* ATCC 29212 strain was reduced to 60% in the number of live cells within 60 min after treatment (Figure 6B). The percentage of live to dead cells in the buffer control treated sample was 94% live cells and 4% dead cells at the end of 60 min. The reduction of live cell density in the BP404 protein treated samples amounted to 9.33%, 20% and 40% by the end of 15 min, 30 min and 45 min after incubation of the samples at 37 °C. The number of cells corresponding to live (green cells) and dead (red cells) was counted in each frame of the picture, and the percentage of the live and dead cells was calculated and is represented as graph on the right side of the confocal microscope acquired image. The statistical variance was calculated by one-way ANOVA (* *p* ≥ 0.05; ** *p* ≤ 0.05). The buffer treated control did not show variance in the data obtained (* *p* ≥ 0.05). The protein treated samples showed statistical variance across the tested time points (** *p* ≤ 0.05). The results obtained with the confocal microscopy was consistent with the results observed with the decrease in OD_600_ as shown in Figure 4 and Figure 5. 

### 2.5. Cell Wall Binding Efficiency of BP404 towards the Tested E. faecalis Strains

**Figure 7:**
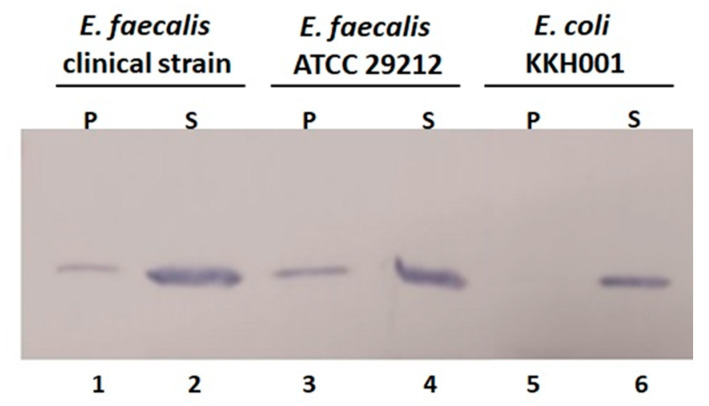
Binding assay to determine the cell wall binding of protein BP404 towards clinical *E. faecalis* and *E. faecalis* ATCC 29212. The protein was found to bind to the bacterial strains and was detected in the pellet fraction (lane 1 and lane 3). Majority of the protein was found as unbound in the supernatant fraction in both clinical *E. faecalis* and *E. faecalis* ATCC 29212 (lane 2 and lane 4). There was no binding of the protein towards *E. coli* KKH001 bacteria (lane 5) and the complete protein was seen in the unbound supernatant fraction (lane 6).

The antibacterial activity of enzybiotics depends on the efficiency of binding the protein to the cell [33]. In order to test the cell binding activity of the purified protein BP404, 600 ng of the protein BP404 was added to 1 × 10^7^ CFU of both the *E. faecalis* strains (clinical isolate and ATCC 29212) and *E. coli* KKH001, which is a clinical isolate. The protein was found to bind to both *E. faecalis* strains. However, a majority of the protein fraction (approx. 80%) was found not to bind to the cells and was seen in the supernatant fraction (Figure 7: lane 2 and lane 4). The protein (approx. 20%) was also seen to bind to the cells as seen in the pellet fraction (Figure 7: lane 1 and 3). As a control the protein binding to *E. coli* KKH001 was also tested. As seen in Figure 7 (lane 5), the protein was not able to bind to *E. coli* KKH001, as no protein was found in the pellet fraction and the total protein was found in the supernatant fraction (Figure 7: lane 6).

## 3. Discussion

In this work, we used the catalytic domain of BacL_1_ encompassing 329 amino acids and linked it with the cell wall binding domain of PlyV12, which encompass 75 amino acids. The strategy of the construction of this was two-fold. Firstly, BacL_1_ was reported to have the presence of the accessory co-factor BacA, to display its antibacterial activity. We speculated that swapping of the cell wall binding domain of BacL_1_ with the cell wall binding domain from PlyV12 would be sufficient to make the chimeric lysin potent against *E. faecalis* strains. Secondly, the cell wall binding epitope of PlyV12 specifically recognizes a unique carbohydrate moiety usually present in the cell wall of *E. faecalis* and *E. faecium*, which would make the chimeric lysin more specific against the pathogenic bacteria harboring the carbohydrate moiety in their cells wall. As evident from the cell wall binding assay, the recombinant lysin BP404 was able to bind to both *E. faecalis* strains tested and no binding was seen towards the tested *E. coli* KKH001 strain. Another feature of this work is that it is the first instance where a chimeric protein was constructed and was found to be active against *E. faecalis* strains, although there has been reports of chimeric proteins displaying activity against other Gram-positive bacteria including *S. aureus* [34]. However, around 80% of BP404 was found not to bind to the *E. faecalis*. The 20% binding was sufficient to cause a detrimental effect of more than 85% and 60% against the clinical isolate of *E. faecalis* and *E. faecalis* ATCC 29212, respectively.

We are not sure why the binding efficiency of BP404 was less towards the tested *E. faecalis* strains. There could be two possible explanations for the observed result. BP404 is an engineered protein with domains from two unrelated proteins linked together. The folding pattern of the protein could mask the active cell wall binding site, rendering it in accessible or only partly accessible to the host carbohydrate epitope in the cell wall. Another possibility is that only the exact 75 amino acid moiety from the C-terminal of PlyV12, encompassing the sh3b domain was used as the cell wall binding domain in BP404. PlyV12 comprises of 314 amino acid residues, of which the catalytic activity (amidase) resides within the first 155 amino acids [30]. This stretch is followed by a possible linker sequence of approximately 84 amino acids. The sh3b domain of 75 amino acids forms at the C-terminal region after the linker sequence. In the constructed BP404 protein, no linker sequence was added between the N-terminal BacL_1_ catalytic domain and the C-terminal sh3b domain from PlyV12. The linker sequence was not added primarily to keep the size of the functional BP404 protein small, although the addition of a linker may substantially improve the cell wall binding and antimicrobial activity of BP404. It has been previously reported that the addition or modification of a linker between the catalytic and cell wall binding domain increased the bactericidal activity in an engineered endolysin, where the catalytic domain was from a streptococcal lysin and the cell wall binding domain from a completely different lysin [35,36]. Even though truncated from its natural counterpart, the cell wall binding domain of PlyV12 used in BP404 retains all the essential features required for a canonical sh3b domain. The sh3b domains usually possess five to seven β-barrel folds, which are usually arranged antiparallel β-sheets, frequently connected by linker residues that varies in composition and length [33]. As shown in Fig. 2, the PlyV12 sh3b domain used in this study possess seven β-sheet structure separated by coil structure.

There are several reports on the development of enzybiotics against Gram-positive bacteria. The recombinant and purified enzybiotics has been tested as antibacterial agents against Gram-positive bacteria [37,38,39,40]. Enzybiotics are primarily developed and tested against the infections caused by ESKAPE group pathogens (*E. faecium*, *S. aureus*, *K. pneumoniae*, *A. baumannii*, *P. aeruginosa*, and *Enterobacter* spp.), which usually escape from the routinely available antibacterial therapies [41,42]. Even though several studies have pointed out the development of chimeric enzybiotics against pathogens such as *S. aureus* [25,28], only a few studies were targeted against *E. faecalis* bacteria [36]. There are reports on various natural lysins that display activity against *E. faecalis* [43,44]. The cell wall binding domain of enzybiotics usually dictates the lytic spectrum by recognizing and binding to specific moieties in the cell wall and the catalytic domain cleave the specific bonds in the peptidoglycan [33]. The peptidoglycan of *Enterococcus* sp. consists of the repeating disaccharide units of MurNAc-GlcNAc (N-acetylmuramic acid-(β1-4)-N-acetylglucosamine) [45]. There are also stem peptides attached to the MurNAc (NAM) through the amino group of L-alanine and the carboxyl group of the D-lactyl moiety of each MurNAc. The stem peptide of *Enterococcus* sp., generally consists of L-Ala-D-Glu-L-Lys-D-Ala-D-Ala. The interpeptide bridge or cross bridges link the adjacent stem peptide strands through a linkage from L-Lys (NH_2_-position 3) in one stem peptide to D-Ala (CO-position 4) in the adjacent stem peptide. In *E. faecalis*, the interpeptide bridge comprises of 2-3 L-Ala residues [46]. Mutational changes in the amino acid sequences may exist in the stem peptide and cross bridges within individual strains and different species [47]. This could explain why a lower antibacterial activity of BP404 was observed with *E. faecalis* ATCC 29212 strain, in comparison with *E. faecalis* clinical strain.

PlyV12 endolysin was earlier reported to possess antibacterial activity against *E. faecalis*, *E. faecium*, staphylococcal and streptococcal strains, primarily due to its efficiency to bind to these bacterial strains. We speculate that BP404 should be also be active against those strains affected by PlyV12. We have done some preliminary studies to check for the binding of BP404 against MRSA strain. BP404 was able to bind to the MRSA strain (~5% of the total protein), albeit at a much lower intensity in comparison with the *E. faecalis* strains and a zymogram analysis with the MRSA peptidoglycan also showed activity. Further studies are being conducted to assess the activity and cell wall binding of BP404 towards other bacterial strains including *E. faecium*, *S. aureus* and *S. pyogenes*. We also envisage further modifying the BP404 protein to make it more specific towards *E. faecalis* bacteria.

## 4. Materials and Methods

### 4.1. Bacterial Strains and Growth Conditions

The *E. faecalis* strains were used to evaluate the antibacterial efficiency of the protein. *E. faecalis* strain ATCC 29212 were a gift from the King Fahad Specialist Hospital, Dammam, Saudi Arabia. The clinical isolate of *E. faecalis* was collected from Buraidah Central Hospital, Buraydah, Saudi Arabia. Cloning of the genes was performed on *E. coli* XL-1 blue bacterial strain. The recombinant protein expression was performed in *E. coli* BL21 (DE3) bacterial strain. The bacterial strains, *E. coli* XL-1 blue and *E. coli* BL21 (DE3) was a gift from Prof. Dr. Udo Blaesi, MFPL, Vienna, Austria. 

All the bacterial strains were grown in Luria Bertani (LB) media/agar (0.5% yeast extract, 1% NaCl, 1% peptone, Micromaster, India) pH: 7.0. The bacteria were propagated under shaking conditions (150 rpm) at 37 °C in an orbital shaking incubator (Deluxe automatic orbital shaker, India) unless otherwise mentioned. 

### 4.2. Software Used for Protein Structure Prediction

The three-dimensional structure of the protein was predicted using the Phyre program [31]. The secondary structure of the protein was predicted by the proteus structure prediction server [32].

### 4.3. PCR Amplification of Genes

The gene fragment encoding for the catalytic domain of BacL_1_ (987 bp) (GenBank: AB271686.1) [21] and the cell wall binding domain of PlyV12 (225 bp) (GenBank: AY581208.1) [30] were synthesized artificially (Synbio Technologies, NJ, USA). The gene fragment was amplified with primer pair BACFP and BACRP for BacL_1_ and PLYFP and PLYRP for PlyV12 as listed on Table 1. The PCR amplification was done using 2× Pfu mastermix (G-Biosciences, St. Louis, Missouri, USA). The conditions used for the amplification of genes is as follows: (94 °C for 5 min, 94 °C for 1 min, 60 °C for 30 s, 72 °C for 1.25 min, 72 °C for 6 min). The amplification was repeated for 35 cycles. The final PCR product of the expected size was checked and confirmed by resolving on a 1% agarose gel.

### 4.4. Gene Cloning and Expression

The gene amplified with primers BACFP and BACRP was purified by gel filtration chromatography (Thermo Fisher Scientific, Waltham, MA, USA). The purified gene fragment was restricted with BamHI/KpnI (Thermo Fisher Scientific, Waltham, MA, USA) and cloned into the expression vector pQE30 under the IPTG inducible promoter, creating the plasmid pQE-BAC (Table 1). The gene fragment amplified with primers PLYFP and PLYRP was also purified by gel filtration chromatography and was restricted with KpnI/PstI. The gene was cloned into pQE-BAC downstream of the BacL1 encoding gene, creating the plasmid pQE-BACPLY (Table 1). The cloning was confirmed by restriction analysis and PCR amplification. Furthermore, gene sequencing was performed to confirm the sequence of the cloned gene. The gene cloned under the IPTG inducible promoter was expressed with 0.5 mM of IPTG at an OD_600_ of 0.4. The induced cultures were incubated at 18 °C for 16 h under shaking conditions. Protein synthesis was evaluated by resolving a small aliquot of the induced culture on a 12% SDA-PAGE gel. The induced culture was centrifuged at 2350× *g* for 40 min and the pellet was stored at −80 °C until further use.

### 4.5. Purification of the Synthesized Protein

The protein was expressed in soluble form. Hence, native purification of the synthesized protein was performed using the protocol from Qiagen (Qiagen, St. Louis, MO, USA). Since the protein was expressed as a fusion protein with 6x Histidine tag, Ni-NTA agarose column (G-Biosciences, St. Louis, MO, USA) was used for purification. Shortly, native lysis buffer (10 mL: 300 mM NaCl, 10 mM imidazole, 50 mM NaH_2_PO_4_ pH 8.0) was added to the centrifuged cell pellet. Chicken egg white lysozyme (0.1 gm/mL) (Research Lab, Mumbai, Maharashtra, India) was added to the cell pellet. The cell pellet was resuspended in the native buffer with lysozyme and the mix was incubated for 30 min in ice. The resuspended mixture was sonicated for 7 min (40% power, 4 s pulse, 4 s off; Biosafer, Zhichunli, Beijing, China). After sonication, the mix was centrifuged for 40 min at 2000× *g* at 4 °C. The supernatant was collected and Ni-NTA agarose beads (1.0 mL) were added followed by incubation for 2 h at room temperature. The Ni-NTA bound proteins were washed with wash buffer (300 mM NaCl, 20 mM imidazole, 50 mM NaH_2_PO_4_ pH 8.0), to remove any unbound proteins. The Ni-NTA proteins were eluted with the elution buffer (300 mM NaCl, 250 mM imidazole, 50 mM NaH_2_PO_4_ pH 8.0). The eluted protein was dialyzed in buffer A (20 mM Tris, 50 mM NaCl, 20 mM MgCl_2_, 0.5% Triton x-100 pH 7.5) at 4 °C for 16 h. The dialyzed protein was assessed for purity by separating in a 12% polyacrylamide gel. The protein concentration was measured with nanodrop (Bio-Rad, Des Plaines, IL, USA) and the protein was stored at −20 °C with addition of 20% glycerol.

### 4.6. Activity Assessment of the Protein against Enterococcus faecalis Strains

In order to test the activity of the purified protein against *E. faecalis*, varying concentrations of the protein ranging from 150 ng to 15,000 ng per ml were added to 1 × 10^7^ CFU of two *E. faecalis* strains in buffer A, pH: 7.5. The bacteria treated with proteins were incubated at 37 °C for 120 min. The OD_600_ of the culture was estimated at different time points with a spectrophotometer (Shimadzu, Japan). The experiment was performed in triplicates and the mean value was taken for calculations.

### 4.7. Live/Dead Staining and Confocal Microscopy

The protein treated bacterial cells along with the control was analyzed for live/dead staining to evaluate the percentage of live and dead cells, as per the protocol described by Manoharadas et al. [48]. In short, SYTO9 (Thermo Fisher Scientific, Waltham, MA, USA) dye (5 μM) was diluted in DMSO and was added to the bacterial cells. The mix was allowed to stay in dark conditions for 10 min. Following incubation for 10 min, the cells were centrifuged at 10,000 rpm for 2 min and the supernatant was discarded. The cell pellet was washed extensively for 3 times in 1× PBS solution and was rinsed with autoclaved distilled water. Propidium iodide (Thermo Fischer Scientific, Waltham, MA, USA) was resuspended to a final concentration of 500 nM in SSC buffer (0.03 M sodium citrate, 0.3 M NaCl, pH 7.0). After addition of the propidium iodide to the cells, the mix was incubated at room temperature for 10 min. The mix was centrifuged and the supernatant was discarded. The pellet was washed extensively three times with 1× PBS, and final washing was performed with autoclaved distilled water. The cells were mounted onto a glass slide and was covered with cover slip without any trapped air bubbles. Imaging of the live (SYTO9 excitation/emission at 483/503 nm) and dead cells (propidium iodide excitation/emission at 535/617 nm) was done with spinning disk confocal microscope (Zeiss, Jena, Germany). The camera used for capturing the images on the confocal microscope was Rolera Em-C^2^ camera with the objective specification of 63 × oil immersion (Zeiss, Jena, Germany). The captured images were processed with the Zen lite software (Zeiss, Jena, Germany). The number of live (green) and dead (red) cells per frame/picture was counted manually. The experiment was performed in triplicates and the mean value was taken for the calculations.

### 4.8. Evaluation of Cell Binding Activity of the Protein

In order to check the potentiality of the recombinant protein to bind to the cell wall of *E. faecalis*, 50 ng of the protein was added to 1 × 10^7^ cells in buffer A, pH 7.5 and incubated at 37 °C for 15 min. After incubation, the mixture was centrifuged at 16,400× *g* for 2 min. The supernatant was withdrawn and the unbound protein was precipitated by the addition of TCA. The cell pellet, following centrifugation, was washed thrice with 1x PBS buffer. The cell pellet was incubated at 94 °C for 10 min after the addition of SDS-PAGE gel loading dye. Both the cell pellet and TCA precipitated protein fraction was resolved on an SDS-PAGE gel and subjected to western blot analysis. The gel resolved proteins were transferred to nitrocellulose membrane (Thermoscientific, USA) by semi dry western blotting apparatus (Bio-rad, USA), for 20 min at 20 V. Blocking of the nitrocellulose membrane after protein transfer was performed at room temperature with the addition of 5% milk powder in 1× TBST buffer for 1 h, shaking conditions (50 rpm). The blocking solution was washed away thrice with 1× TBST buffer. Primary antibody (mouse anti-his antibody, Abclonal, Woburn, Massachusetts, USA) was added to the blot in a dilution of 1:2500 (stock concentration: 0.2 mg/mL; Diluted concentration used: 80 ng/mL) and incubation was performed at 16 °C for 15 h. After incubation, the blot was washed thrice with 1× TBST buffer to remove any nonspecific residual primary antibody. Secondary antibody (goat anti-mouse IgG linked to alkaline phosphatase, Elabscience, Houston, Texas, USA) was further added to the blot in a dilution of 1:12,000 (stock concentration: 0.2 mg/mL; Working concentration: 16.66 ng/mL) and incubated for 1 h at room temperature under shaking conditions (50 rpm). The blot was thoroughly washed thrice with 1× TBST buffer. The development of the blot was performed by the addition of 10 mL of BCIP/NBT (G-Biosciences, USA) substrate. The experiment was performed in triplicate.

### 4.9. Software Used for Graph Preparation Statistical Analysis of the Data

The graphical representation of the acquired data was generated using Microsoft Excel (2010 version). The standard deviation and mean values for the data set was calculated with Microsoft Excel (2010 version). Error bars in the graph represent the standard deviation. The statistical variation of data and *p*-values were calculated by the one-way ANOVA calculator (https://goodcalculators.com/one-way-anova-calculator/ (5 February 2023).

## 5. Conclusions

The development of new antimicrobial agents is critical in the fight against antibiotic resistant bacteria. Although there have been reports of the efficacy of phage proteins against *E. faecalis*, no major studies have dealt with the development of novel chimeric proteins against *E. faecalis*. Herein, we constructed a protein by fusing the catalytic domain from the bacteriocin BacL_1_ with the cell wall binding domain from the phage ϕ1 endolysin PlyV12. The constructed protein BP404 was active against the clinical *E. faecalis* and *E. faecalis* ATCC 29212 strains. We are confident that this work will pave the way for the development of other chimeric proteins against *E. faecalis* bacteria. 

## Figures and Tables

**Table 1:** Primers and plasmids used for the study.

Gene Amplified	Primer Name	Primer Sequence	PCR Product Size (bp)
BacL_1_-Catalytic domain	BACFP	5′AATTCGGATCCATGAATTACAG TCAAAAAGCAATCG3′	987
BacL_1_-Catalytic domain	BACRP	5′ATTCGGTACCATTCACTGAATCTCC TTTTGAACCAGA3′	987
PlyV12- Cell wall binding domain	PLYFP	5′GACCGGTACCGGATTTACGAAGGAA GAAGCTA3′	225
PlyV12-Cell wall binding domain	PLYRP	5′TTACCTGCAGTTACTTAAATGTAC CCCATGCTTCC3′	225
**Plasmid Name**		**Notes on the plasmid**	**Synthetized protein**
pQE30		Expression vector. Induction of promoter with IPTG. N-terminal 6X His-tag for purification.	NA
pQE-BAC		The catalytic domain encoding gene from BacL1 cloned as BamHI/KpnI into pQE30 vector.	NA
pQE-BACPLY		The 225 bp cell wall binding gene from PlyV12 of phage ϕ1 was cloned as KpnI/PstI into pQE-BAC	46 kDa

## Data Availability

The data presented in this study are available on reasonable request from the corresponding author.

## Supplementary Materials

The following supporting information can be downloaded at: https://www.mdpi.com/article/10.3390/antibiotics12020388/s1, Figure S1: Calculated *p*-values for text Figure 4 and Figure 5.
