# Supplementary material for: Construction and Activity Testing of a Modular Fusion Peptide against Enterococcus faecalis
Source: Antibiotics (Basel). 2023 Feb 14;12(2):388. doi: 10.3390/antibiotics12020388 (PMC9951850; doi:10.3390/antibiotics12020388)
Supplement: Supplementary file 1 [file antibiotics-12-00388-s001.zip › antibiotics-2172031-supplementary.pdf]

Figure S1: Calculated *p*-values for text Figures 4 and 5

Buffer-treated control *E. faecalis* ATCC 29212

Analysis of Variance Results

F-statistic value = 1.93294

P-value = 0.16219

Time (min)	Groups	N	Mean	Std. Dev.	Std. Error
	**Data Summary**				
0	Group 1	3	100	0	0
15	Group 2	3	91.7376	7.0592	4.0757
30	Group 3	3	88.2754	5.1134	2.9522
45	Group 4	3	87.446	6.566	3.7909
60	Group 5	3	87.5339	9.2092	5.3169
120	Group 6	3	85.9954	6.8225	3.9389

**ANOVA Summary**					
Source	Degrees of Freedom DF	Sum of Squares SS	Mean Square MS	F-Stat	P-Value
**Between Groups**	5	403.4163	80.6833	1.9329	0.1622
**Within Groups**	12	500.8948	41.7412		
**Total:**	17	904.3111			

BP404/150 ng + *E. faecalis* ATCC 29212

Analysis of Variance Results

F-statistic value = 21.47908

P-value = 0.00001

Time (min)	Data Summary				
	Groups	N	Mean	Std. Dev.	Std. Error
0	Group 1	3	100	0	0
15	Group 2	3	92.5531	4.5019	2.5991
30	Group 3	3	85.2549	4.0799	2.3555
45	Group 4	3	80.2306	4.4532	2.5711
60	Group 5	3	74.4624	4.3486	2.5107
120	Group 6	3	69.3766	5.7551	3.3227

ANOVA Summary					
Source	Degrees of Freedom DF	Sum of Squares SS	Mean Square MS	F-Stat	P-Value
**Between Groups**	5	1946.9926	389.3985	21.4791	0
**Within Groups**	12	217.5504	18.1292		
**Total:**	17	2164.543			

BP404/600 ng + *E. faecalis* ATCC 29212

Analysis of Variance Results

F-statistic value = 70.06407

P-value = 0

Time (min)	Data Summary				
	Groups	N	Mean	Std. Dev.	Std. Error
0	Group 1	3	100	0	0
15	Group 2	3	82.2077	2.2513	1.2998
30	Group 3	3	74.3752	3.3666	1.9437
45	Group 4	3	70.105	3.6677	2.1175
60	Group 5	3	66.0796	2.7927	1.6124
120	Group 6	3	63.6175	2.9626	1.7105

ANOVA Summary					
Source	Degrees of Freedom DF	Sum of Squares SS	Mean Square MS	F-Stat	P-Value
**Between Groups**	5	2710.9274	542.1855	70.0641	0
**Within Groups**	12	92.8611	7.7384		
**Total:**	17	2803.7885			

BP404/5400 ng + *E. faecalis* ATCC 29212

Analysis of Variance Results

F-statistic value = 315.20925

P-value = 0

Time (min)	Groups	N	Mean	Std. Dev.	Std. Error
	Data Summary				
0	Group 1	3	100	0	0
15	Group 2	3	90.9645	2.5508	1.4727
30	Group 3	3	80.3174	3.3285	1.9217
45	Group 4	3	71.579	2.2369	1.2915
60	Group 5	3	67.1205	1.4951	0.8632
120	Group 6	3	39.3696	0.9426	0.5442

ANOVA Summary					
Source	Degrees of Freedom DF	Sum of Squares SS	Mean Square MS	F-Stat	P-Value
**Between Groups**	5	6754.1549	1350.831	315.2092	0
**Within Groups**	12	51.4261	4.2855		
**Total:**	17	6805.581			

BP404/15000 ng + *E. faecalis* ATCC 29212

Analysis of Variance Results

F-statistic value = 106.46001

P-value = 0

Time (min)	Data Summary				
	Groups	N	Mean	Std. Dev.	Std. Error
0	Group 1	3	100	0	0
15	Group 2	3	80.7189	3.897	2.25
30	Group 3	3	70.98	4.792	2.7667
45	Group 4	3	64.2004	4.0891	2.3608
60	Group 5	3	50.6748	4.5494	2.6266
120	Group 6	3	39.3257	1.802	1.0404

ANOVA Summary					
Source	Degrees of Freedom DF	Sum of Squares SS	Mean Square MS	F-Stat	P-Value
**Between Groups**	5	6992.192	1398.4384	106.46	0
**Within Groups**	12	157.6297	13.1358		
**Total:**	17	7149.8217			

Buffer-treated control *E. faecalis* clinical strain

Analysis of Variance Results

F-statistic value = 0.55765

P-value = 0.73056

Time (min)	Groups	N	Mean	Std. Dev.	Std. Error
	**Data Summary**				
0	Group 1	3	100	0	0
15	Group 2	3	91.4761	8.6021	4.9664
30	Group 3	3	97.5701	14.1413	8.1645
45	Group 4	3	89.5609	3.4852	2.0122
60	Group 5	3	95.9443	7.4142	4.2806
120	Group 6	3	99.2237	15.4817	8.9383

Source	Degrees of Freedom DF	Sum of Squares SS	Mean Square MS	F-Stat	P-Value
**ANOVA Summary**					
**Between Groups**	5	269.8896	53.9779	0.5576	0.7306
**Within Groups**	12	1161.545	96.7954		
**Total:**	17	1431.4346			

BP404/150 ng + *E. faecalis* clinical strain

Analysis of Variance Results

F-statistic value = 493.56562

P-value = 0

Time (min)	Data Summary				
	Groups	N	Mean	Std. Dev.	Std. Error
0	Group 1	3	100	0	0
15	Group 2	3	88.4184	0.7278	0.4202
30	Group 3	3	71.3492	2.0561	1.1871
45	Group 4	3	54.3454	0.9997	0.5772
60	Group 5	3	45.5076	3.9297	2.2688
120	Group 6	3	38.2311	0.8837	0.5102

ANOVA Summary					
Source	Degrees of Freedom DF	Sum of Squares SS	Mean Square MS	F-Stat	P-Value
**Between Groups**	5	9040.5213	1808.1043	493.5656	0
**Within Groups**	12	43.9602	3.6634		
**Total:**	17	9084.4815			

BP404/600 ng + *E. faecalis* clinical strain

Analysis of Variance Results

F-statistic value = 131.76728

P-value = 0

Time (min)	Data Summary				
	Groups	N	Mean	Std. Dev.	Std. Error
0	Group 1	3	100	0	0
15	Group 2	3	89.3856	5.5946	3.2301
30	Group 3	3	76.7851	4.1918	2.4201
45	Group 4	3	61.9264	3.9308	2.2695
60	Group 5	3	47.637	2.8466	1.6435
120	Group 6	3	40.686	1.5974	0.9222

ANOVA Summary					
Source	Degrees of Freedom DF	Sum of Squares SS	Mean Square MS	F-Stat	P-Value
Between Groups	5	8232.9015	1646.5803	131.7673	0
Within Groups	12	149.9535	12.4961		
Total:	17	8382.855			

BP404/5400 ng + *E. faecalis* clinical strain

Analysis of Variance Results

F-statistic value = 323.73242

P-value = 0

Time (min)	Groups	N	Mean	Std. Dev.	Std. Error
	**Data Summary**				
0	Group 1	3	100	0	0
15	Group 2	3	93.3465	0.8546	0.4934
30	Group 3	3	78.1829	2.5638	1.4802
45	Group 4	3	64.52	3.3284	1.9216
60	Group 5	3	45.8622	4.5681	2.6374
120	Group 6	3	31.075	1.0657	0.6153

Source	Degrees of Freedom DF	Sum of Squares SS	Mean Square MS	F-Stat	P-Value
**ANOVA Summary**					
**Between Groups**	5	10894.9211	2178.9842	323.7324	0
**Within Groups**	12	80.7698	6.7308		
**Total:**	17	10975.6909			

BP404/15000 ng + *E. faecalis* clinical strain

Analysis of Variance Results

F-statistic value = 391.42536

P-value = 0

Time (min)	Data Summary				
	Groups	N	Mean	Std. Dev.	Std. Error
0	Group 1	3	100	0	0
15	Group 2	3	93.8344	3.7144	2.1445
30	Group 3	3	77.5195	3.9508	2.281
45	Group 4	3	54.7234	2.5946	1.498
60	Group 5	3	32.3017	3.3614	1.9407
120	Group 6	3	13.7329	2.7213	1.5712

ANOVA Summary					
Source	Degrees of Freedom DF	Sum of Squares SS	Mean Square MS	F-Stat	P-Value
**Between Groups**	5	17888.7981	3577.7596	391.4254	0
**Within Groups**	12	109.684	9.1403		
**Total:**	17	17998.4821			
